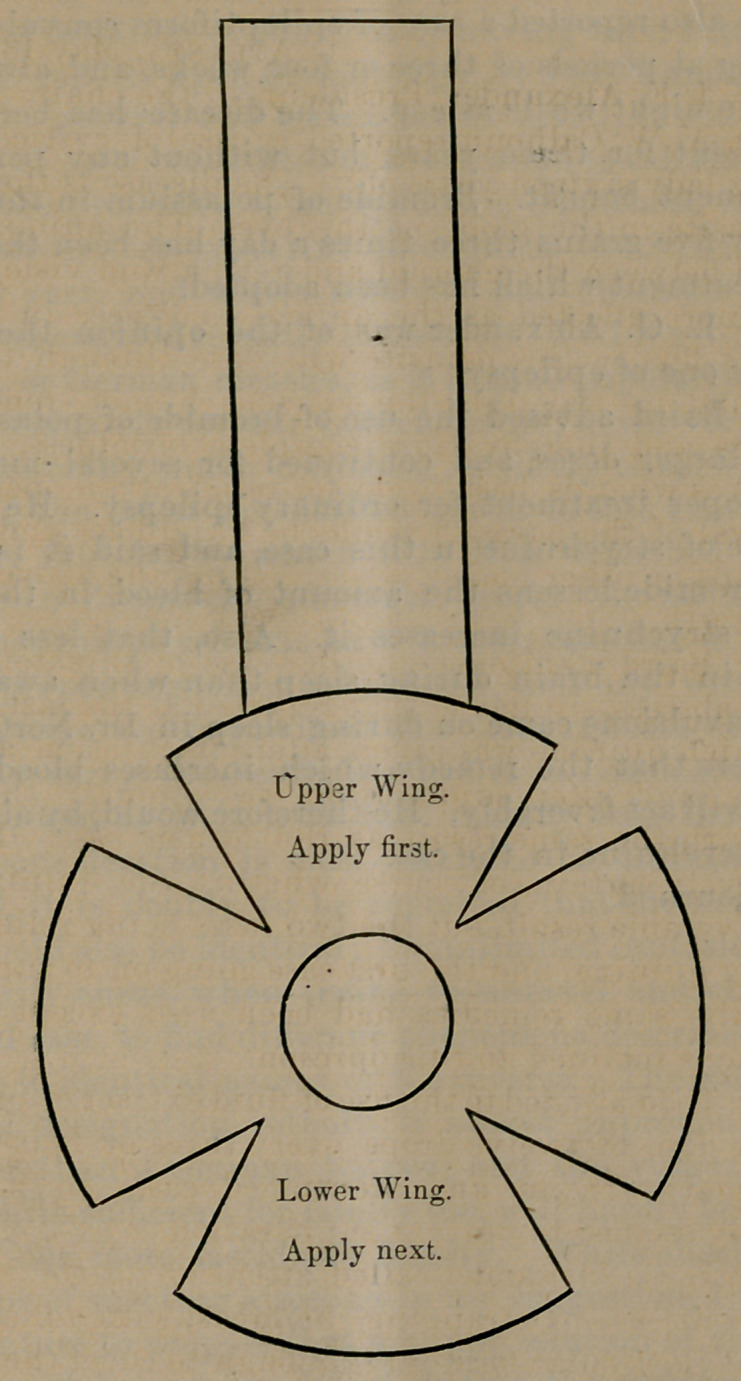# Atlanta Academy of Medicine

**Published:** 1878-06

**Authors:** J. G. Westmoreland

**Affiliations:** Reporter


### Reports of Societies.

### ATLANTA ACADEMY OF MEDICINE.

J. G. WESTMORELAND, M.D., REPORTER.

ATLANTA, GA., April 15, 1878.

Dr. J. T. Johnson, Vice-President, in the chair.

Dr. J. G. Westmoreland reported the case of a young lady who is suffering from partial paralysis of the right lower extremity with hyperœsthesia of the dorsum of the foot. The difficulty has existed for seven months, and was, according to the history received from the patient, preceded by erysipelatous inflammation of the leg and foot. Several years previously she had an attack of typhoid fever, which left her with slight numbness and inactivity of both thumbs; the left was restored in a short time, but the right thumb has never regained its natural feeling. The patient does not limp very perceptibly, but finds great difficulty in walking much during the day, from apparent weakness of the muscles concerned in locomotion. No organic or functional disease of abdominal or pelvic viscera could be detected by which the nervous difficulty could be accounted for by reflex impression.

The treatment instituted consists of electric and strychnine stimulation of the spinal nervous system and means to improve the appetite, which is poor, and to give improvement to the digestion. For these purposes interrupted galvanic or faradic current daily, one thirty-second of a grain of strychnine three times a day, with muriated tincture of iron and pancreo-peptine occasionally. The preparation of iron is given more with the view of obtaining the tonic effect of the acid upon the stomach, to increase the appetite and digestion, than for any effect of iron upon the blood or nervous system; both of which, however, may be of some consideration.

Dr. Baird said the case would certainly receive benefit from electricity, and that he should certainly continue that means. In such cases he thinks the faradic decidedly preferable to the uninterrupted galvanic current. He thinks the shocks of the former more powerful upon the muscles, and causing them more forcibly to contract, is decidedly more useful in paralysis than the continued current of galvanism. He thinks favorably of strychnine in gradually increased doses until the effect is perceptible in the muscular system.

Dr. Baird reported a case of mammary abscess which was treated with fomentations, anodynes, etc., but finally, from accumulation of pus, required lancing. Subsequently she had symptoms of a return, and the breast, being treated as before, did not improve, when the treatment by quinine was adopted, and successfully in preventing suppuration.

He also reported a case of diseased thumb in a female who had suffered with neglected felon, resulting in death of the distal phalanx, keeping up constant discharge throuh small openings. The patient objecting very positively to the use of the knife, the necrosed bone was effectually removed with forceps through an opening at the end of the thumb.

---

ATLANTA, May 13, 1878.

Dr. J. F. Alexander, President, in the chair.

Dr. A. W. Calhoun reported a case of cysticercus in a young lady sixteen years old. The disease is the result of a parasite in the globe of the eye. The animal was detected between the choroid and retina, and vision is more and more affected as the animal increases in size. He thinks finally the parasite will bore through the retina and find its way into the vitreous humor, rendering it opaque, and thereby cause complete blindness. Dr. C. is at a loss what is best to do for relief, as an operation for removal of the parasite would be hazardous, and there is perhaps no other means of preventing the above result of its growth.

Dr. Baird mentioned another case of threatened mammary abscess, similar to that reported at the last meeting, in which quinine was given after fomentation and other means, and after suppuration was strongly threatened, and with the same result as the former case. He is not positively certain, of course, whether the quinine led to the favorable result, but the two cases being relieved after taking quinine, and the first case going on to suppuration after the same remedies had been used except the quinine, he is inclined to this opinion.

Dr. Todd alluded to the use of fluid extract of phptolacca in the dose of twelve drops every three or four hours, in mammary abscess, and mentioned cases in which the remedy seemed to prevent suppuration.

Dr. L. G. Alexander called attention to the treatment of this disease by strapping the breast with adhesive plaster cut in circular form large enough to cover the mamma. In the center of this a hole is made large enough for the mamma to pass through. Four segments are to be cut in the circle one inch wide and coming to a point two-thirds of the distance to the central hole for the nipple. The breast being emptied of two-thirds the milk, the upper portion, which has attached a strap two inches wide passing over the shoulder, is made to adhere, and then the lower portion, so as to give firm pressure and support, and then the lateral wings.

This treatment he also uses successfully in preventing accumulation of milk when no nursing can be had. He has used it with relief also in enlarged mammary glands at the menstrual period.

Dr. North, of Senoia, being present by invitation, gave a succinct history of a case of stone in the bladder, in which rapid recovery occurred after extraction of the stone by the bilateral operation.

He also reported a case of epileptiform convulsions, occurring at periods of three or four weeks, and always during the night while asleep. The disease has been under treatment for three years, but without any perceptible permanent benefit. Bromide of potassium in the dose of twenty-five grains three times a day has been the principal treatment which has been adopted.

Dr. L. G. Alexander was of the opinion the case is clearly one of epilepsy.

Dr. Baird advised the use of bromide of potassium in much larger doses, and continued for several months, as the proper treatment for ordinary epilepsy. He advised the use of strychnine in this case, and said it is known that bromide lessens the amount of blood in the brain, while strychnine increases it. Also, that less blood is found in the brain during sleep than when awake. As the convulsions came on during sleep in Dr. North's case, he infers that the remedy which increases blood in the brain will act favorably. He therefore would, by all means, give strychnine in the case.

Adjourned.

## Figures and Tables

**Figure f1:**